# Factors associated with the self-esteem of pregnant women at normal risk

**DOI:** 10.1590/0034-7167-2024-0264

**Published:** 2025-03-31

**Authors:** Caroline Bessa da Silva, Victórya Suéllen Maciel Abreu, Douglas de Araújo Costa, Luisa Gomes Viana, Priscila de Souza Aquino, Camila Biazus Dalcin, Ana Karina Bezerra Pinheiro, Arlene de Jesus Mendes Caldas

**Affiliations:** I Universidade Federal do Ceará. Fortaleza, Ceará, Brazil; II Universidade de Dundee. Dundee, Scotland, United Kingdom; III Universidade Federal do Maranhão. São Luís, Maranhão, Brazil

**Keywords:** Self Concept, Pregnant Women, Association, Nursing, Health., Autoimagen, Mujeres Embarazadas, Asociación, Enfermería, Salud.

## Abstract

**Objectives:**

to analyze the factors associated with the self-esteem of normal risk pregnant women.

**Methods:**

this is a cross-sectional study conducted with 150 pregnant women at normal risk from three primary health care units in the city of Fortaleza, Ceará, Brazil. Two instruments were used: a questionnaire (sociodemographic, gynecological-obstetric history and current pregnancy) and the Rosenberg Self-Esteem Scale. Student’s t-test was used for data analysis.

**Results:**

it was observed that the factors associated with low self-esteem of pregnant women were age up to 19 years (p=0.023), not being married (p=0.005), not living with a partner (p=0.049), not having a paid occupation during pregnancy (p=0.026) and not planning the pregnancy (p=0.044).

**Conclusions:**

pregnant adolescents, not having a partner and without pay affect their self-esteem. These aspects should be considered a priority for investment in health, management and organization of health services.

## RESUMEN

**Objetivos:**

analizar los factores asociados a la autoestima de gestantes de riesgo normal.

**Métodos:**

estudio transversal realizado con 150 gestantes de riesgo normal de tres unidades de atención primaria de salud de la ciudad de Fortaleza, Ceará, Brasil. Se utilizaron dos instrumentos: un cuestionario (sociodemográfico, antecedentes gineco-obstétricos y embarazo actual) y la Escala de Autoestima de Rosenberg. Para el análisis de los datos se utilizó la prueba t de Student.

**Resultados:**

se observó que los factores asociados a la baja autoestima de las gestantes fueron la edad hasta 19 años (p=0,023), no estar casada (p=0,005), no vivir con pareja (p=0,049), no tener una ocupación remunerada durante el embarazo (p=0,026) y no planificar el embarazo (p=0,044).

**Conclusiones:**

las adolescentes embarazadas, no tener pareja y no tener remuneración afectan su autoestima. Estos aspectos deben considerarse una prioridad para la inversión en salud, gestión y organización de los servicios de salud.

## INTRODUCTION

Pregnancy represents a phase of rapid biopsychosocial changes, with potential positive or negative impacts on women’s lives. The myriad issues encompassing pregnancy, childbirth, and early childhood care present significant challenges for families and healthcare services, given the complexity of the demands involved^(1)^. Among these challenges, the influence of pregnancy on women’s self-esteem stands out, often intertwined with physical transformations such as weight gain and emotional fluctuations during pregnancy and the postpartum period, which may have repercussions on body image^(2)^.

Self-esteem, recognized as a fundamental psychological need, is defined as an individual’s self-assessment shaped by attitudes, emotions, feelings, perspectives, and thoughts cultivated through life experiences and interactions with others^(3)^.

Regarding low self-esteem during pregnancy, it is essential to underscore the heightened vulnerability to depression. Studies have established associations between sudden weight gain during pregnancy, birthing experiences, stress, and the prevalence of depression and low self-esteem among women^(2,4,5)^. Additionally, data from the French “Eden” cohort reveal that children exposed to maternal prenatal depression and anxiety are at a higher risk of following more problematic socio-emotional and behavioral trajectories throughout childhood^(6)^.

Primary factors contributing to the development of low self-esteem among pregnant women include lower educational attainment, unemployment, and the occurrence of cesarean sections^(7)^. Moreover, other factors may also be implicated, underscoring the necessity of conducting studies to comprehensively evaluate self-esteem during pregnancy and its associated determinants^(8)^.

Recognizing the global imperative for a positive pregnancy experience entails prioritizing the maintenance of both physical and sociocultural health, ensuring the well-being of both mother and baby throughout the pregnancy journey, facilitating a smooth transition to labor and birth, and ultimately fostering positive motherhood experiences. Central to this framework are considerations of maternal self-esteem, competence, and autonomy^(9)^.

In this context, the importance of comprehensive prenatal care cannot be overstated in the pursuit of positive pregnancy experiences. Prioritizing discussions on this matter is essential as it equips healthcare professionals with the ability to effectively pinpoint factors contributing to low self-esteem during pregnancy. This heightened awareness enables better monitoring and facilitates the provision of a more positive experience throughout this critical phase^(10)^. Consequently, the significance of addressing psychological aspects, particularly maternal self-esteem, in prenatal consultations becomes evident.

## OBJECTIVES

To analyze the factors associated with the self-esteem of pregnant women at normal risk.

## METHODS

### Ethical aspects

The study was conducted in accordance with national and international ethical guidelines and was approved by the Research Ethics Committee of the Federal University of Ceará, Brazil. The guidelines described in Resolution 466/12 of the National Health Council on studies involving human beings were followed. Written informed consent was obtained from all individuals involved in the study.

### Type of study, period and location of study

This is a cross-sectional study conducted with pregnant women at normal risk from three primary health units located in the city of Fortaleza, Ceará, Brazil, from May 2022 to May 2023. The three primary health units were selected for convenience. All units belonged to the same health region, serving a similar population.

As the study employed a cross-sectional design, the research process, from data collection to final writing, was guided by the application of the Strengthening the Reporting of Observational Studies in Epidemiology (STROBE) tool^(11)^.

### Study Sample

The sample was selected by convenience. The inclusion criteria included women over 18 years of age without gestational risk and with a gestational age greater than 20 weeks. A study showed that negative body attitudes increased progressively throughout pregnancy^(12)^. Another study indicated that 54.6% of women with 21 weeks or more had unsatisfactory self-esteem, despite a lack of direct association^(13)^. Therefore, this cutoff point was used in sample selection. Exclusion criteria included pregnant women unable to respond to the interview due to mental, neurological, or communication problems.

A sample size calculation was conducted, taking into account a confidence level of 95% (1.96), a margin of error of 0.8 points on the scale, and a standard deviation of 4.75 obtained from a previous study conducted with Brazilian pregnant women^(14)^. This calculation yielded a sample size of 136 pregnant women. To account for potential data loss, an additional 14 participants were included, resulting in a final sample size of 150 pregnant women.

### Instruments Used

The data collection instrument was administered to the convenience sample according to the inclusion and exclusion criteria. Two instruments were used in the study: one comprising a sociodemographic questionnaire, gynecological-obstetric history, and data on the current pregnancy, and the other being the Rosenberg Self-Esteem Scale (RSES). The sociodemographic questionnaire included nine questions related to gynecological-obstetric history, 14 items on the current pregnancy, and 15 questions addressing sociodemographic factors.

The RSES, developed by Morris Rosenberg in 1965, was translated and validated in Brazil by Gal Dini in 2004, yielding a Cronbach’s Alpha of 0.90. This scale provides a comprehensive assessment of an individual’s positive or negative self-perception. It comprises 10 items rated on a Likert scale, with five items reflecting positive orientation (1, 3, 4, 7, and 10) and five reflecting negative orientation (2, 5, 6, 8, and 9)^(15)^.

Respondents rate their agreement with each statement on a scale from “strongly disagree” to “strongly agree”, with each option assigned a numerical value. The total score represents the self-esteem level of the research subject, with scores ranging from 0 to 3 per statement. A higher overall score indicates lower self-esteem^(15)^.

### Data collection

Nursing students administered the data collection instruments in a private office adjacent to the prenatal care area, either before or after the consultation. After reading, clarifying, and signing the informed consent form, participants completed the instruments in private, with the process typically lasting about 15 minutes.

The predictor variables included age, place of origin, ethnicity, marital status, cohabitation with a partner, religion, educational attainment, employment status during pregnancy, total number of pregnancies, history of abortion, complications during the current pregnancy, frequency of prenatal consultations, pregnancy planning, and cohabitation status. The primary outcome variable measured the score on the scale assessing personal self-esteem.

### Analysis of results and statistics

Data analysis was conducted using the Statistical Package for the Social Sciences (SPSS) software version 20.0. Descriptive statistics were used to summarize numerical variables, including absolute and relative frequencies, means, medians, and standard deviations. Student’s t-test for independent samples was employed, and the Shapiro-Wilk test assessed the normality of the variables. Statistical significance was defined as p<0.05.

## RESULTS

The study sample consisted of pregnant women aged 18 to 40, with a median age of 27 years. The median monthly income was R$ 1,212.00, approximately $248.49, with incomes ranging from R$ 250.00 to R$ 10,000.00 (equivalent to $51.22 to $2,048.63). The women had an average of 11 years of education, ranging from one to 19 years. Most identified as having brown ethnicity (110; 73.3%) and resided in the capital, Fortaleza (137; 91.3%). A substantial portion reported being married (53; 35.3%) and living with their partner (122; 81.3%).

In terms of clinical and obstetric characteristics, there was a prevalence of multiple pregnancies (101; 67.3%) and multiparous women (94; 62.7%). Cesarean section was the predominant mode of delivery (51; 54.3%). The median number of prenatal consultations was five, ranging from one to 13. Notably, a significant proportion of pregnant women reported unplanned pregnancies (105; 70.0%), and a majority (85; 56.7%) did not attend prenatal appointments accompanied by their partners. Table 1 presents a comparative analysis of sociodemographic factors and scores on the RSES.

**Table 1:** Comparative analysis of mean Rosenberg Self-Esteem Scale scores across sociodemographic factors, Fortaleza, Ceará, Brazil, 2023, N=150

Sociodemographic factors	MATERNAL SELF-ESTEEM					
	n	%	Mean	Mean difference	IC of the difference	*p* ^*^
Age						
Up to 19 years old	12	8.0	11.67	3.457	0.494 - 6.419	**0.023**
Over 19 years old	138	92.0	8.21			
Region of origin						
Capital	137	91.3	8.71	2.554	(-0.323) - (5.432)	0.081
Countryside	13	8.7	6.15			
Income						
Up to 1 salary	78	52.0	8.82	0.696	(-0.955) - (2.346)	0.406
Above 1 salary	72	48.0	8.13			
Education						
Up to 9 years	37	24.7	9.73	1.650	(-0.228) - (3.529)	0.085
Over 9 years	113	75.3	8.08			
Marital status						
Married	53	35.3	7.04	-2.241	(-3.800) - (-0.681)	**0.005**
Other	97	64.7	9.28			
Religion						
Yes	118	78.7	8.08	-1.884	(-3.857) - (0.089)	0.061
No	32	21.3	9.97			
Ethnicity						
Brown	110	73.3	8.27	-0.802	(-2.647) - (1.043)	0.392
Other	40	26.7	9.08			
Lives with partner						
Yes	122	81.3	8.10	-2.080	(-4.152) - (-0.008)	**0.049**
No	28	18.7	10.18			
Paid employment during pregnancy						
Yes	77	51.3	7.60	-1.827	(-3.437) - (-0.218)	**0.026**
No	73	48.7	9.42			

* *Student's t-test.*

Statistical significance was observed between age and maternal self-esteem, with adolescents aged up to 19 years (p=0.023) exhibiting lower maternal self-esteem compared to older women. Similarly, pregnant women who were not married demonstrated lower self-esteem than married women (p=0.005), as did pregnant women who did not cohabit with their partners (p=0.049). Additionally, pregnant women without paid employment during this period (p=0.026) displayed higher scores on the scale, indicative of lower self-esteem. Table 2 compares the means of clinical and obstetric factors with the RSES score.

**Table 2:** Comparative analysis of mean Rosenberg Self-Esteem Scale scores across clinical and obstetric factors, Fortaleza, Ceará, Brazil, 2023, N=150

Clinical and obstetric factors	MATERNAL SELF-ESTEEM					
	n	%	Mean	Mean difference	IC of the difference	*p* ^*^
Number of pregnancies						
Primigravida	49	32.7	8.12	-0.541	(-2.283) - (1.201)	0.558
Multigravida	101	67.3	8.66			
Route of last delivery (n=89)						
Cesarean	48	53.9	9.29	1.292	(-0.810) - (3.393)	0.225
Vaginal	41	46.1	8.00			
Previous Miscarriage (n=101)						
Yes	29	28.7	8.69	0.037	(-2.133) - (2.206)	0.973
No	72	71.3	8.65			
Planned pregnancy						
Yes	45	30.0	7.22	-1.806	(-3.567) - (-0.046)	**0.044**
No	105	70.0	9.03			
Number of prenatal visits						
Up to 7	127	84.7	8.75	1.705	(-0.549) - (3.958)	0.137
More than 7	23	15.3	7.04			
Companion in consultations						
Yes	65	43.3	8.09	0.696	(-2.343) - (0.951)	0.405
No	85	56.7	8.79			
Complications in current pregnancy						
Yes	15	10	8.47	-0.022	(-2.749) - (2.704)	0.987
No	135	90	8.49			

* *Student's t-test.*

In terms of clinical and obstetric factors, a noteworthy correlation was found between pregnancy planning and maternal self-esteem (p=0.044). This finding indicates that women who did not plan their pregnancy exhibited lower levels of self-esteem compared to those who had planned it.

## DISCUSSION

Self-esteem encompasses not only perceptions of body image, appearance, or physical attractiveness but also emotional attitudes toward one’s body^(3)^. A study conducted with 287 pregnant women in Poland aimed to analyze the factors influencing body self-esteem and its correlation with self-efficacy. It was observed that older and more educated women tended to evaluate their bodies more favorably^(16)^. These findings parallel those uncovered in the current study, where pregnant women aged 18 and 19 exhibited lower maternal self-esteem compared to their older counterparts.

Regarding geographical origin, it is imperative to underscore the influence of sociocultural aspects specific to a region on maternal self-esteem. Women often face societal pressures to conform to appearance-related ideals, a phenomenon exacerbated during pregnancy due to the myriad physical, psychological, and physiological changes experienced. Such pressures can lead to body dissatisfaction and psychological distress^(17)^. In the present study, no statistical significance was found in the analysis between women residing in urban or rural areas. However, research conducted in São Paulo, Brazil, involving 264 women, revealed that those residing in major urban centers are more susceptible to stress, social comparison, and low self-esteem^(18)^. Thus, new social dynamics within urban environments also play a role in shaping self-esteem.

Furthermore, it is crucial to acknowledge the significant and direct correlation between sociodemographic factors and psychosocial themes during pregnancy, particularly in relation to self-esteem. A study involving 417 women receiving care in both public and private healthcare sectors in Brazil demonstrated that those served by the public sector tended to have lower levels of education and socioeconomic status compared to their counterparts in the private sector. Additionally, a significant association was observed between low self-esteem and negative body attitudes. Notably, demographic disparities were more pronounced among pregnant women accessing care through the public health system, underscoring the need for heightened attention to psychological factors that may often go overlooked^(19)^. Therefore, it is reasonable to infer that pregnant women receiving care in the public sector require greater psychological support, as these factors may not always be adequately addressed.

In terms of vulnerabilities, the absence of support from a partner can precipitate low self-esteem, reluctance toward pregnancy, and even depressive symptoms, adversely impacting the quality of life for pregnant women^(20)^. In the present study, unmarried women who did not live with their partners had lower self-esteem. This phenomenon was similarly observed in a study involving 131 pregnant women in South Korea, highlighting the detrimental effects of inadequate support on prenatal depression and low self-esteem^(21)^. Hence, it can be inferred that maternal psychological well-being is closely linked to the positive reinforcement derived from a supportive partner, shedding light on the potential reasons for low self-esteem among single pregnant women.

Although religion was not shown to be related in the present study, this variable has been associated with other outcomes related to self-esteem. In pregnant women, studies have observed its relationship with lower anxiety^(22)^ and fewer negative effects of stress^(23)^.

Furthermore, in this study, the lack of paid employment was related to low self-esteem. In Turkey, pregnant women of lower socioeconomic status exhibited higher depression scores and lower self-esteem. A study involving 385 pregnant women revealed that those with paid employment and higher levels of education reported elevated self-esteem^(10)^. Thus, understanding the social context and addressing potential vulnerabilities among pregnant women can lead to improvements in self-esteem.

This perspective is corroborated by evidence indicating lower levels of self-esteem among unemployed women, alongside a heightened prevalence of other issues such as anxiety^(24)^ and perinatal depressive symptoms^(25)^. This aligns with a global trend suggesting that women lacking paid employment tend to experience lower self-esteem, potentially due to socioeconomic vulnerabilities, given that employment often correlates with improved financial circumstances.

In the obstetric context, a study conducted in Finland involving 125 women revealed a direct correlation between a positive birth experience and higher self-esteem during the first year postpartum^(4)^. This suggests that both the mode of the last birth and the overall pregnancy experience can significantly influence maternal self-esteem.

In the present study, there was no statistical significance regarding previous vaginal or cesarean births, but women who underwent cesarean sections reported lower maternal self-esteem. This finding is consistent with research aimed at evaluating risk factors contributing to postpartum depression, which suggests that a high rate of cesarean sections may predispose individuals to postpartum depression due to increased stress, somatic complaints, and low self-esteem^(26)^.

It is evident, therefore, that low self-esteem during pregnancy is multifactorial. Among the contributing factors is unplanned pregnancy. A study involving 225 women from Ecuador highlighted lower self-esteem among those whose pregnancies were unplanned^(27)^. France’s national survey found higher odds of perceived poor psychological health among women whose pregnancy was unwelcomed^(28)^. A study carried out in the south of the Netherlands with 1928 pregnant women showed that women with an unplanned pregnancy (N = 111, 5.8%) reported persistently higher levels of depressive symptoms during the entire perinatal period compared to women with a planned pregnancy, after adjustment for confounders (p < 0.001)^(25)^.

Thus, it can be surmised that women who did not plan their pregnancies may feel unprepared to cope with the changes that occur during this period, consequently experiencing challenges with their self-esteem.

### Study limitations

This study has limitations that should be considered. Firstly, the recruitment of participants was limited to the city of Fortaleza, in the state of Ceará, Brazil, and the small sample size may restrict the generalization of our results. Future studies should include pregnant women from other regions and expand the sample size.

### Contributions to Nursing

It is worth highlighting the importance of furthering study in the nursing field, as nursing professionals play a fundamental role in prenatal care. Through the factors evidenced in this study, nurses can promote ways to improve maternal self-esteem and thus foster a positive experience during pregnancy.

## CONCLUSIONS

The study identified several factors associated with low self-esteem in pregnant women, including being aged up to 19 years, being unmarried, not cohabiting with a partner, lacking employment during pregnancy, and experiencing unplanned pregnancies. Therefore, it is imperative to emphasize the importance of assessing maternal self-esteem, especially within the context of sociodemographic and obstetric vulnerabilities that influence this aspect. In light of these findings, implementing targeted healthcare interventions for pregnant women becomes crucial. These interventions should aim to support women in overcoming challenges that could potentially hinder a positive pregnancy experience.
