# Supplementary material for: Factors associated with the self-esteem of pregnant women at normal risk
Source: Rev Bras Enferm. 2025 Mar 31;78(2):e20240264. doi: 10.1590/0034-7167-2024-0264 (PMC11961045; doi:10.1590/0034-7167-2024-0264)
Supplement: Translation

# Translation: Fatores associados à autoestima de gestantes de risco habitual

**DOI:** 10.1590/0034-7167-2024-0264pt

**Authors:** Caroline Bessa da Silva, Victórya Suéllen Maciel Abreu, Douglas de Araújo Costa, Luisa Gomes Viana, Priscila de Souza Aquino, Camila Biazus Dalcin, Ana Karina Bezerra Pinheiro, Arlene de Jesus Mendes Caldas

**Affiliations:** I Universidade Federal do Ceará. Fortaleza, Ceará, Brasil; II Universidade de Dundee. Dundee, Escócia, Reino Unido; III Universidade Federal do Maranhão. São Luís, Maranhão, Brasil

**Keywords:** Autoimagem, Gestantes, Associação, Enfermagem, Saúde.

## Abstract

**Objetivos:**

analisar os fatores associados à autoestima de gestantes.

**Métodos:**

estudo transversal com 150 gestantes de risco habitual em Fortaleza, Ceará, de maio de 2022 a maio de 2023. Utilizados dois instrumentos de coleta, um de caracterização sociodemográfica, antecedentes gineco-obstétricos e dados da gestação atual e a Escala de Autoestima de Rosenberg, para análise foi realizado o Teste T de Student para amostras independentes, todos assinaram o Termo de Consentimento Livre e Esclarecido com aprovação pelo Comitê de Ética em Pesquisa da Universidade Federal do Ceará.

**Resultados:**

os fatores associados à baixa autoestima de gestantes foram idade até 19 anos (p=0,023), não ser casada (p=0,005), não residir com o parceiro (p=0,049), não possuir ocupação remunerada durante a gestação (p=0,026) e não planejar a gravidez (p=0,044).

**Conclusões:**

sendo assim, a avaliação da autoestima materna é primordial em um cenário de vulnerabilidades sociodemográficas e obstétricas que afetam esse desfecho.

## INTRODUÇÃO

A gestação é uma fase de mudanças biopsicossociais que ocorrem em um curto intervalo de tempo, podendo trazer repercussões positivas ou negativas para a vida das mulheres. As diferentes questões envolvidas na gestação, no parto e na primeira infância da criança tornam esse período do ciclo de desenvolvimento um desafio para as famílias e para a assistência realizada pelos serviços de saúde, devido à complexidade das demandas que acarreta^(1)^. Um dos aspectos que podem ser influenciados pela gestação é a autoestima das mulheres, que pode estar relacionada a mudanças que ocorrem no corpo durante a gravidez e o pós-parto, como o ganho de peso e as alterações de humor, ocasionando consequências para a imagem corporal da mulher^(2)^.

A autoestima, além de ser considerada uma necessidade, pode ser definida como uma avaliação que se faz de si mesmo por meio de atitudes, emoções, sentimentos, perspectivas e pensamentos que o próprio indivíduo adquire ao longo da vida, como consequência das interações com outras pessoas e eventos^(3)^.

Com relação à baixa autoestima na gestação, é válido destacar a maior vulnerabilidade à depressão. Estudos evidenciam que o aumento súbito de peso durante a gravidez, as experiências de parto e o estresse estão relacionados com a depressão e a baixa autoestima nas mulheres^(2,4,5)^. Além disso, dados da coorte francesa “Eden” revelam que crianças expostas à depressão e à ansiedade pré-natal materna tiveram um risco maior de seguir trajetórias socioemocionais e comportamentais mais problemáticas ao longo da infância^(6)^.

Os principais fatores que levam as gestantes a desenvolverem baixa autoestima relacionam-se à baixa escolaridade, à falta de ocupação e a cirurgias cesarianas^(7)^. Ademais, outros fatores podem estar associados, o que torna imprescindível a realização de estudos que avaliem a autoestima no período gestacional e seus fatores associados^(8)^.

Destaca-se a valorização mundial da ocorrência de uma experiência positiva de gravidez, definida como a manutenção da normalidade da saúde física e sociocultural, de uma gravidez saudável para a mãe e o bebê, da transição efetiva para o trabalho de parto positivo e nascimento e do alcance da maternidade positiva (incluindo autoestima materna, competência e autonomia)^(9)^.

Nesse contexto, sabe-se que a adequação da assistência pré-natal é indispensável para alcançar os objetivos propostos de experiência positiva na gestação. É importante que esse tema seja debatido e que os profissionais de saúde saibam identificar os fatores relacionados à baixa autoestima na gestação para um melhor acompanhamento e provisão de uma experiência positiva nesse período^(10)^. Percebe-se, assim, a relevância de abordar aspectos psicológicos nas consultas de pré-natal, incluindo a autoestima materna.

## OBJETIVOS

Analisar os fatores associados à autoestima de gestantes de risco habitual.

## MÉTODOS

### Aspectos éticos

A pesquisa foi aprovada pelo Comitê de Ética em Pesquisa da Universidade Federal do Ceará, Brasil. Foram respeitadas as recomendações da Resolução nº 466/12 do Conselho Nacional de Saúde para estudos envolvendo seres humanos. O Termo de Consentimento Livre e Esclarecido (TCLE) foi assinado por todos os envolvidos no estudo.

### Tipo de estudo, período e local do estudo

Trata-se de um estudo transversal realizado com gestantes de risco habitual de três unidades de saúde primária situadas no município de Fortaleza, Ceará, Brasil, no período de maio de 2022 a maio de 2023. As três unidades básicas de saúde foram selecionadas por conveniência com base na área de atuação do programa de internato de enfermagem da universidade.

Além disso, por se tratar de um estudo transversal, utilizou-se a ferramenta Strengthening the Reporting of Observational Studies in Epidemiology (STROBE), que objetiva melhorar a metodologia dos estudos e, por conseguinte, os resultados^(11)^.

### Amostra do Estudo

A amostra foi constituída por conveniência. Foram incluídas mulheres sem risco gestacional, maiores de 18 anos e com idade gestacional superior a 20 semanas. Um estudo mostrou que as atitudes corporais negativas aumentaram progressivamente ao longo da gestação^(12)^. Além disso, outro estudo mostrou um percentual de 54,6% de autoestima insatisfatória em mulheres com 21 semanas ou mais, apesar da ausência de associação^(13)^. Portanto, decidiu-se utilizar esse ponto de corte na seleção da amostra. Os critérios de exclusão incluíram gestantes que não conseguiram responder à entrevista devido a problemas mentais ou neurológicos, bem como problemas de comunicação.

Foi realizado cálculo amostral, considerando um nível de confiança de 95% (1,96), um erro de 0,8 pontos na escala, bem como o desvio-padrão de 4,75 obtido em estudo anterior realizado com gestantes brasileiras^(14)^, resultando em uma amostra de 136 gestantes. Esse valor foi acrescido de 14 gestantes para correção de eventuais perdas de dados, tornando a amostra final deste estudo composta por 150 gestantes.

### Instrumentos Utilizados

O instrumento de coleta de dados foi aplicado em amostras selecionadas por conveniência de acordo com os critérios de inclusão e exclusão. Foram aplicados dois instrumentos: um para a caracterização sociodemográfica, antecedentes gineco-obstétricos e dados da gestação atual, e a Escala de Autoestima de Rosenberg. O questionário sociodemográfico contém 9 questões relacionadas aos antecedentes gineco-obstétricos, 14 itens sobre a gravidez atual e 15 perguntas direcionadas a questões sociodemográficas.

A escala de avaliação de autoestima - Rosenberg Self-Esteem Scale (RSES) de Morris Rosenberg (1965) - foi traduzida e validada no Brasil, com Alfa de Cronbach de 0,90, por Gal Dini em 2004. Ela avalia globalmente a atitude positiva ou negativa do indivíduo em relação a si mesmo. A RSES é constituída por 10 itens, em uma escala tipo Likert, com cinco itens de orientação positiva (1, 3, 4, 7 e 10) e cinco itens de orientação negativa (2, 5, 6, 8 e 9)^(15)^.

A RSES é composta de opções que variam de “discordo totalmente” a “concordo totalmente”. Para esta versão, a cada opção de resposta é atribuído um valor, e a soma dos valores correspondentes à resposta de cada item resulta no desempenho de cada sujeito da pesquisa. Cada frase pode receber um escore mínimo de 0 e máximo de 3. Quanto maior o escore obtido na escala, menor o nível de autoestima^(15)^.

### Coleta de dados

Os estudantes de enfermagem aplicaram os instrumentos de coleta de dados em um consultório fechado, próximo ao local onde o pré-natal era realizado, antes ou depois da consulta. Após a leitura, esclarecimento e assinatura do termo de consentimento livre e esclarecido, os participantes preencheram os instrumentos em sigilo, um processo que durava, em média, 15 minutos.

As variáveis preditoras foram: idade, procedência, etnia, estado civil, viver com um parceiro, religião, escolaridade, ter ocupação remunerada durante a gestação, total de gestações, aborto, intercorrências durante a gestação atual, número de consultas, gravidez planejada e se vivia com parceiro. A variável desfecho foi a pontuação da escala relacionada à autoestima pessoal.

### Análise dos resultados e estatística

Os dados foram tabulados e analisados através do software Statistical Package for Social Sciences (SPSS), versão 20.0. Para variáveis numéricas, foram atribuídos valores absolutos e relativos, médias, medianas e desvio padrão. Foi realizado o teste T de Student para amostras independentes. A condição de normalidade das variáveis foi avaliada pelo teste de Shapiro-Wilk. Valores de p<0,05 indicaram significância estatística.

## RESULTADOS

Observou-se que as gestantes tinham idade entre 18 e 40 anos, com mediana de 27 anos, renda mediana de R$ 1.212,00 (aproximadamente $248,49), com valor mínimo de R$ 250,00 e máximo de R$ 10.000,00 (entre $51,22 e $2048,63). Quanto à escolaridade, verificou-se uma média de 11 anos de estudo, variando de um a 19 anos. Houve predomínio da etnia parda, com 110 participantes (73,3%), procedentes da capital Fortaleza, 137 (91,3%), sendo a maioria casada, 53 (35,3%) e vivendo com o parceiro, 122 (81,3%).

Quanto aos fatores clínicos e obstétricos, houve predominância de multigestas, 101 (67,3%), multíparas, 94 (62,7%), e a última via de parto predominante foi a cesárea (51; 54,3%). O número de consultas realizadas apresentou mediana de cinco consultas, variando de uma a 13. Houve também predominância de gestantes que não planejaram a gravidez, 105 (70,0%). Além disso, observou-se que 85 (56,7%) mulheres não levavam o companheiro às consultas de pré-natal.

Na Tabela 1, verifica-se a comparação das médias dos fatores sociodemográficos com a pontuação da Escala de Autoestima de Rosenberg.

**Tabela 1:** Comparação entre as médias da pontuação da Escala de Autoestima de Rosenberg, segundo os fatores sociodemográficos, Fortaleza, Ceará, Brasil, 2023, N=150

Fatores Sociodemográficos	AUTOESTIMA MATERNA					
	n	%	Média	Diferença de Média	IC da diferença	*p* ^*^
Idade						
Até 19 anos	12	8,0	11,67	3,457	0,494 - 6,419	**0,023**
Acima de 19 anos	138	92,0	8,21			
Procedência						
Capital	137	91,3	8,71	2,554	(-0,323) - (5,432)	0,081
Interior	13	8,7	6,15			
Renda						
Até 1 salário	78	52,0	8,82	0,696	(-0,955) - (2,346)	0,406
Acima de 1 salário	72	48,0	8,13			
Escolaridade						
Até 9 anos	37	24,7	9,73	1,650	(-0,228) - (3,529)	0,085
Acima de 9 anos	113	75,3	8,08			
Estado Civil						
Casada	53	35,3	7,04	-2,241	(-3,800) - (-0,681)	**0,005**
Outros	97	64,7	9,28			
Religião						
Sim	118	78,7	8,08	-1,884	(-3,857) - (0,089)	0,061
Não	32	21,3	9,97			
Etnia						
Parda	110	73,3	8,27	-0,802	(-2,647) - (1,043)	0,392
Outras	40	26,7	9,08			
Vive com parceiro						
Sim	122	81,3	8,10	-2,080	(-4,152) - (-0,008)	**0,049**
Não	28	18,7	10,18			
Ocupação remunerada durante a gestação						
Sim	77	51,3	7,60	-1,827	(-3,437) - (-0,218)	**0,026**
Não	73	48,7	9,42			

* *Teste t de Student.*

Observa-se uma significância estatística entre a idade e a autoestima materna, constatando-se que, em média, adolescentes com até 19 anos (p=0,023) têm uma autoestima materna menor que as mulheres mais velhas. Da mesma forma, gestantes que não são casadas apresentam autoestima menor do que as casadas (p=0,005), assim como gestantes que não residem com o parceiro (p=0,049). Além disso, gestantes sem ocupação remunerada durante esse período (p=0,026) apresentam maiores pontuações na escala, o que reflete uma menor autoestima.

Na Tabela 2, verifica-se a comparação das médias dos fatores clínicos e obstétricos com a pontuação da Escala de Autoestima de Rosenberg.

**Tabela 2:** Comparação entre médias da pontuação da Escala de Autoestima de Rosenberg, segundo os fatores clínicos e obstétricos, Fortaleza, Ceará, Brasil, 2023, N=150

Fatores Clínicos e Obstétricos	AUTOESTIMA MATERNA					
	n	%	Média	Diferença de Média	IC da diferença	*p* ^*^
Número de gestações						
Primigesta	49	32,7	8,12	-0,541	(-2,283) - (1,201)	0,558
Multigesta	101	67.3	8.66			
Última via de parto (n=89)						
Cesariana	48	53,9	9,29	1,292	(-0,810) - (3,393)	0,225
Vaginal	41	46,1	8,00			
Aborto Anterior (n=101)						
Sim	29	28,7	8,69	0,037	(-2,133) - (2,206)	0,973
Não	72	71.3	8.65			
Gravidez Planejada						
Sim	45	30,0	7,22	-1,806	(-3,567) - (-0,046)	**0,044**
Não	105	70,0	9,03			
Número de Consultas Pré-Natal						
Até 7	127	84,7	8,75	1,705	(-0,549) - (3,958)	0,137
Mais de 7	23	15,3	7,04			
Companheiro nas Consultas						
Sim	65	43,3	8,09	0,696	(-2,343) - (0,951)	0,405
Não	85	56,7	8,79			
Intercorrências na Gestação atual						
Sim	15	10	8,47	-0,022	(-2,749) - (2,704)	0,987
Não	135	90	8,49			

* *Teste t de Student.*

Quanto às variáveis clínicas e obstétricas, observou-se uma associação significativa entre o planejamento da gravidez e a autoestima materna (p=0,044), demonstrando que mulheres que não planejaram a gravidez possuíam uma autoestima menor que as mulheres que a planejaram.

## DISCUSSÃO

A autoestima materna não está relacionada apenas à percepção da imagem corporal, aparência ou atratividade física, mas também às atitudes emocionais em relação ao corpo ^(3)^. Em estudo realizado com 287 grávidas na Polônia, que objetivou analisar fatores que influenciam a autoestima corporal e sua relação com a autoeficácia, foi observado que mulheres mais velhas e com maior escolaridade avaliaram melhor seus corpos^(16)^. Tais achados assemelham-se aos encontrados no presente estudo, no qual gestantes de 18 e 19 anos apresentaram uma autoestima materna menor que as mulheres mais velhas.

Em relação à procedência, é importante destacar a influência dos aspectos socioculturais de determinada região na autoestima materna. As mulheres são constantemente expostas à pressão sociocultural para atender aos ideais de aparência. Na gestação, com as mudanças físicas, psicológicas e fisiológicas, essa pressão pode provocar insatisfação corporal e sofrimento psicológico^(17)^. No presente estudo, não foi constatada significância estatística na análise entre as mulheres que moravam na capital ou no interior. No entanto, um estudo realizado em São Paulo, Brasil, com 264 mulheres, demonstrou que as mulheres que vivem em grandes centros urbanos estão mais propensas ao estresse, à comparação social e à baixa autoestima^(18)^. Dessa forma, novas interações sociais na cidade passam a influenciar também a constituição da autoestima.

Além disso, é importante destacar que fatores sociodemográficos são fundamentais e estão diretamente relacionados a aspectos psicossociais na gestação, como a autoestima. Um estudo realizado com 417 mulheres atendidas em setores públicos e privados no Brasil demonstrou que mulheres atendidas no setor público tinham menor escolaridade e níveis socioeconômicos inferiores em comparação com aquelas atendidas pelo setor privado. Evidenciou-se também uma associação significativa entre baixa autoestima e atitudes corporais negativas. No entanto, os fatores demográficos foram mais preocupantes entre as gestantes atendidas no setor público de saúde quando comparadas às da rede privada^(19)^. Dessa forma, é válido inferir que gestantes atendidas no setor público necessitam de maior atenção quanto a fatores psicológicos que, em muitos casos, não são avaliados.

Ainda no que se refere às vulnerabilidades, a falta de apoio do companheiro pode desencadear baixa autoestima, recusa da gravidez e até sintomas depressivos, o que repercute negativamente na qualidade de vida da gestante^(20)^. No presente estudo, mulheres solteiras e que não moravam com seus companheiros apresentaram menor autoestima. Essa realidade também foi observada em um estudo com 131 mulheres grávidas na Coreia do Sul, que demonstrou a influência da falta de apoio na depressão pré-natal e na baixa autoestima^(21)^. Pode-se inferir, portanto, que o bem-estar psicológico materno está relacionado com o reforço positivo da convivência com o companheiro, o que pode justificar a baixa autoestima nas gestantes solteiras.

Embora a religião não tenha mostrado relação no presente estudo, essa variável tem sido associada a outros desfechos relacionados à autoestima. Em gestantes, estudos observaram sua relação com menor ansiedade^(22)^ e com menos efeitos negativos do estresse^(23)^.

Além disso, neste estudo, a falta de emprego remunerado foi associada à baixa autoestima. Na Turquia, mulheres com menor nível socioeconômico apresentaram maiores escores de depressão e menor autoestima. Um estudo desenvolvido com 385 mulheres demonstrou que aquelas com ocupação remunerada e ensino superior apresentaram autoestima elevada^(10)^. Assim, compreender a situação social e reduzir as possíveis vulnerabilidades das gestantes contribuirá para o aumento da autoestima.

Essa perspectiva é confirmada, pois mulheres desempregadas tendem a apresentar níveis mais baixos de autoestima, além de maior ocorrência de outros agravos, como ansiedade^(24)^ e sintomas depressivos perinatais^(25)^. Isso confirma a tendência mundial de que mulheres sem ocupação remunerada têm menor autoestima, o que pode estar relacionado à vulnerabilidade socioeconômica, considerando que a ocupação é sinônimo de melhores condições financeiras.

Quanto ao contexto obstétrico, uma pesquisa realizada na Finlândia com 125 mulheres demonstrou que, quanto mais positiva for a experiência do parto, maior a autoestima no primeiro ano pós-parto^(4)^. Dessa forma, é possível afirmar que a via do último parto e a experiência da gestante podem interferir na autoestima materna.

No presente estudo, não houve significância estatística quanto à realização anterior de parto vaginal ou cesárea, mas as mulheres que realizaram o parto cesárea tinham menor autoestima materna. Isso corrobora um estudo que objetivou avaliar os fatores de risco que contribuem para a depressão pós-parto, afirmando que o alto índice de cesáreas pode predispor à depressão pós-parto, por meio do aumento do estresse, das queixas somáticas e da baixa autoestima^(26)^.

Fica claro, portanto, que a baixa autoestima na gestação é multicausal. Entre os fatores, destaca-se a gravidez não planejada. Em um estudo realizado com 225 mulheres no Equador, observou-se que a autoestima foi menor entre mulheres cuja gravidez não foi planejada^(27)^. Uma pesquisa nacional na França descobriu que, entre mulheres com gestações não planejadas, havia maior chance de percepção de saúde psicológica ruim, especialmente entre as mulheres cuja gravidez não foi bem-vinda^(28)^. Um estudo realizado no sul da Holanda com 1.928 mulheres grávidas mostrou que aquelas com uma gravidez não planejada (N = 111, 5,8%) relataram níveis persistentemente mais altos de sintomas depressivos durante todo o período perinatal em comparação com mulheres com uma gravidez planejada, após ajuste para fatores de confusão (p < 0,001)^(25)^.

Essa realidade é semelhante à observada no presente estudo. Pode-se entender que as mulheres que não planejaram a gravidez podem sentir-se despreparadas para lidar com as mudanças que ocorrem nesse período, repercutindo em problemas de autoestima.

### Limitações do estudo

Este estudo apresenta limitações que devem ser consideradas. Em primeiro lugar, o recrutamento dos participantes foi limitado à cidade de Fortaleza, no estado do Ceará, Brasil, e o pequeno tamanho da amostra pode restringir a generalização dos resultados. Estudos futuros devem incluir gestantes de outras regiões e aumentar o tamanho da amostra.

### Contribuições para a Área

É válido destacar a importância do aprofundamento do estudo para a área da enfermagem, visto que o profissional de enfermagem tem papel fundamental na assistência pré-natal e pode, com base nos fatores evidenciados neste estudo, promover formas de melhorar a autoestima materna e, assim, proporcionar uma experiência positiva na gestação.

## CONCLUSÕES

O estudo permitiu identificar que os fatores associados à baixa autoestima de gestantes foram idade até 19 anos, não ser casada, não residir com o parceiro, não possuir ocupação remunerada durante a gestação e não ter planejado a gravidez.

Dessa forma, é importante ressaltar que a avaliação da autoestima materna é primordial, especialmente em um cenário de vulnerabilidades sociodemográficas e obstétricas que afetam esse desfecho. Nesse contexto, torna-se cabível a aplicação de ações de saúde com enfoque nas gestantes, de modo a direcioná-las para resolver impasses que podem dificultar uma experiência positiva na gestação.
